# Modulation of Autophagy by a Thioxanthone Decreases the Viability of Melanoma Cells

**DOI:** 10.3390/molecules21101343

**Published:** 2016-10-10

**Authors:** Raquel T. Lima, Diana Sousa, Ana M. Paiva, Andreia Palmeira, João Barbosa, Madalena Pedro, Madalena M. Pinto, Emília Sousa, M. Helena Vasconcelos

**Affiliations:** 1 i3S-Instituto de Investigação e Inovação em Saúde, Universidade do Porto, Rua Alfredo Allen 208, 4200-135 Porto, Portugal; rlima@ipatimup.pt (R.T.L.); dsousa@ipatimup.pt (D.S.); 2 Cancer Drug Resistance Group, IPATIMUP—Institute of Molecular Pathology and Immunology of the University of Porto, Rua Júlio Amaral de Carvalho, 45, 4200-135 Porto, Portugal; 3 Department of Pathology, FMUP—Faculty of Medicine of the University of Porto, Alameda Prof. Hernâni Monteiro, 4200-319 Porto, Portugal; 4 Laboratory of Microbiology, Department of Biological Sciences, FFUP—Faculty of Pharmacy, University of Porto, Rua de Jorge Viterbo Ferreira 228, 4050-313 Porto, Portugal; 5 Laboratory of Organic and Pharmaceutical Chemistry, Department of Chemical Sciences, FFUP—Faculty of Pharmacy, University of Porto, Rua de Jorge Viterbo Ferreira 228, 4050-313 Porto, Portugal; gpaivamafalda@gmail.com (A.M.P.); andreiapalmeira@gmail.com (A.P.); madalena@ff.up.pt (M.M.P.); esousa@ff.up.pt (E.S.); 6 CESPU, Instituto de Investigação e Formação Avançada em Ciências e Tecnologias da Saúde, IUCS—Instituto Universitário de Ciências da Saúde, Rua Central de Gandra 1317, 4585-116 Gandra, Portugal; j.filipebarbosa@hotmail.com (J.B.); madalena.oliveira.pedro@gmail.com (M.P.); 7 CIIMAR/CIMAR—Centro Interdisciplinar de Investigação Marinha e Ambiental, Universidade do Porto, Terminal de Cruzeiros do Porto de Leixões, Avenida General Norton de Matos, S/N, 4450-208 Matosinhos, Portugal

**Keywords:** thioxanthones, melanoma, autophagy, cell death, apoptosis

## Abstract

(1) Background: Our previous studies unveiled the hit thioxanthone TXA1 as an inhibitor of P-glycoprotein (drug efflux pump) and of human tumor cells growth, namely of melanoma cells. Since TXA1 is structurally similar to lucanthone (an autophagy inhibitor and apoptosis inducer) and to *N*^10^-substituted phenoxazines (isosteres of thioxanthones, and autophagy inducers), this study aimed at further assessing its cytotoxic mechanism and evaluating its potential as an autophagy modulator in A375-C5 melanoma cells; (2) Methods: Flow cytometry with propidium iodide (PI) for cell cycle profile analysis; Terminal deoxynucleotidyl transferase dUTP nick end labeling (TUNEL) assay, flow cytometry with Annexin V/PI labeling and Western blot for apoptosis analysis were conducted. A pharmacophore approach was used for mapping TXA1 onto pharmacophores for autophagy induction. Autophagy analyses included transmission electron microscopy for visualization of autophagic structures, fluorescence microscopy for observation of monodansylcadaverine (MDC) staining, pattern of LC3 expression in the cells and acridine orange staining, and Western blot for autophagic proteins expression; (3) Results: TXA1 induced autophagy of melanoma cells at the GI_50_ concentration (3.6 μM) and apoptosis at twice that concentration. Following treatment with TXA1, autophagic structures were observed, together with the accumulation of autophagosomes and the formation of autophagolysosomes. An increase in LC3-II levels was also observed, which was reverted by 3-methyladenine (3-MA) (an early stage autophagy-inhibitor) but further increased by E-64d/pepstatin (late-stage autophagy inhibitors). Finally, 3-MA also reverted the effect of TXA1 in cellular viability; (4) Conclusion: TXA1 decreases the viability of melanoma cells by modulation of autophagy and may, therefore, serve as a lead compound for the development of autophagy modulators with antitumor activity.

## 1. Introduction

Autophagy is a catabolic process which targets cellular organelles and cytoplasmic constituents to the lysosomes for degradation, allowing the cell to maintain homeostasis and being particularly relevant during nutrient deprivation and other stresses [1]. Alterations in autophagy are frequently found in diseases, such as cancer [2], and might be relevant for cancer response to therapy. Although autophagy has been traditionally regarded as a pro-survival mechanism, it has also been related to cell death [2,3]. In fact, a cross-talk between autophagy and apoptosis has been documented, with these pathways sharing various mediators [4,5,6].

The effect of autophagy in cancer therapy is still under debate and may depend on several factors, such as the cellular context, as well as on the levels and duration of cellular autophagy [7,8]. Indeed, targeting autophagy may provide new opportunities for cancer drug discovery. Several small molecules which modulate autophagy have been already described [8,9,10]. Interestingly, promotion of cell death for cancer therapy has been observed not only with autophagy inducers, but also with autophagy inhibitors [11].

The use of autophagy inhibitors, such as chloroquine, hydroxychloroquine, and lucanthone, in association with chemotherapeutic agents, has already reached clinical trials [7]. On the other hand, the use of small molecules that promote autophagy has also been shown to induce cell death in cancer cells [8]. Indeed, resveratrol was shown to induce cell death in several human tumor cell lines by triggering both autophagy and apoptosis [12,13,14]. In addition, imatinib (Gleevec), a known inducer of apoptosis, was shown to activate the cellular autophagy machinery in mammalian cell lines, including chronic myelogenous leukemia cells [15]. Furthermore, tetrahydrocannabinol was reported as an inducer of cell death by activating autophagy in glioma cells [16]. Likewise, curcumin [17], as well as some of its derivatives [18,19,20], were shown to activate autophagy in human cancer cell lines. Additionally, a novel small molecule (STF-62247) was shown to promote autophagic cell death in Von Hippel Lindau (*VHL*)-deficient renal cell carcinoma cells [21]. Finally, phenethyl isothiocyanate suppressed the Akt/mTOR pathway in human prostate cancer cell lines, leading to several autophagic features [22].

In previous studies from our group, the thioxanthone TXA1 has emerged as a hit compound, modulating P-glycoprotein activity in chronic myeloid leukemia cells [23], inhibiting human tumor cell growth and inducing apoptosis [23,24]. This compound has been shown to be particularly potent in melanoma cells [24].

Since the mechanism of action of this hit molecule is still not fully understood and since TXA1 was structurally similar to molecules known to modulate autophagy (lucanthone and to *N*^10^-substituted phenoxazines) [25,26], this study aimed at further assessing the cytotoxic mechanism of TXA1 in A375-C5 melanoma cells, particularly at further evaluating its potential as an autophagy modulator.

## 2. Results

### 2.1. Treatment of A375-C5 Melanoma Cells with Twice the GI_50_ Concentration of TXA1 Alters the Cell Cycle Profile and Induces Apoptosis Although No Effect Is Found with the GI_50_ Concentration

**Figure 1:**
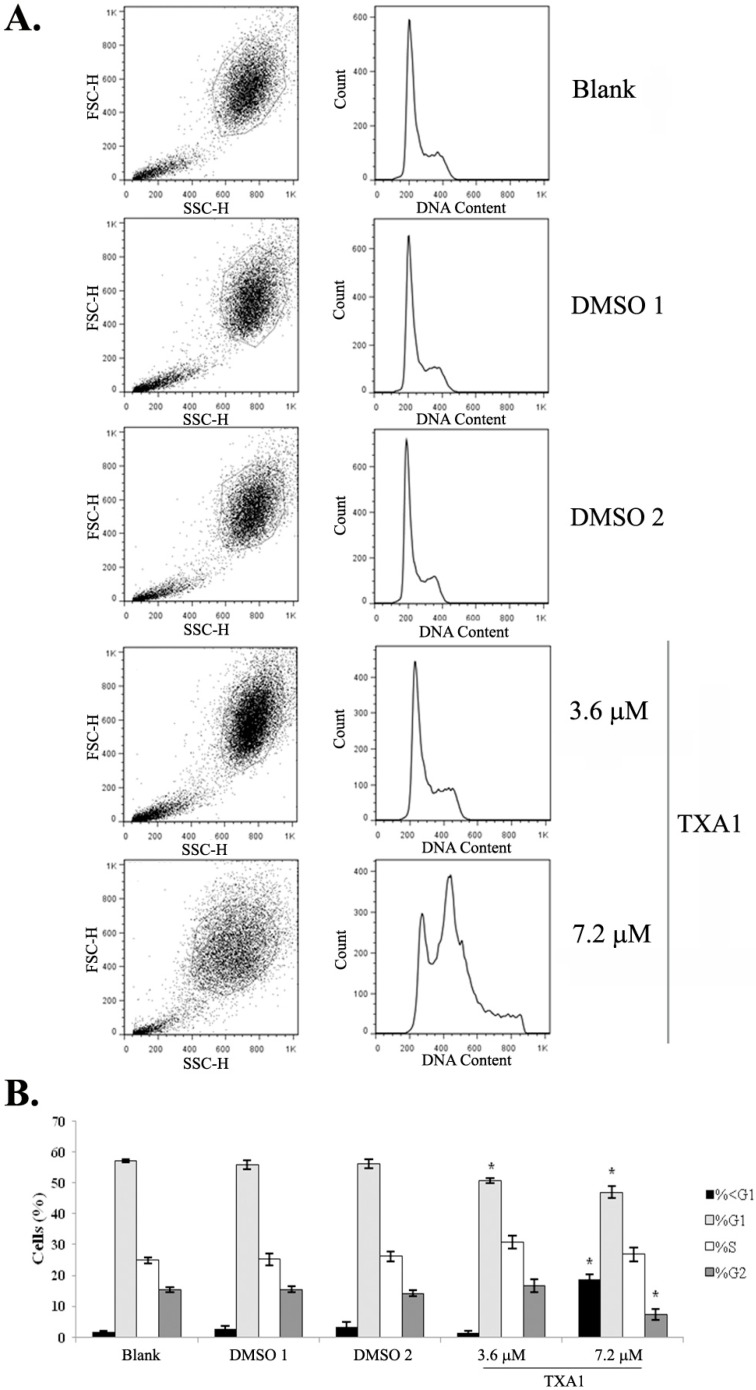
The effect of TXA1 on the cell cycle profile of A373-C5 cells. Cells were treated for 48 h with medium (blank), with TXA1 (3.6 μM and 7.2 μM), or with the corresponding DMSO concentrations (DMSO 1 and DMSO 2, respectively). (**A**) Representative images of flow cytometry analyzed following DNA staining with propidium iodide (PI). Left panels correspond to dot plots of forward vs. side scatter (FSC vs. SSC) and show the gated population. Right panels correspond to the histograms with cell cycle profile of the gated population, following the exclusion of cellular aggregates and debris (data not shown); (**B**) The percentage of cells in the different cell cycle phases. Results are the mean ± SEM of four independent experiments. * *p* < 0.05 blank vs. treatment. Etoposide (2 μM) was used as positive control: G0/G1: 6.3% ± 2.0%; S: 10.4% ± 2.3%; G2/M: 78.3% ± 0.7%.

The effect of TXA1 on the A375-C5 cell cycle profile was analyzed, testing two concentrations of this molecule: the previously-determined GI_50_ concentration (3.6 μM) [24] and twice this concentration (2 × GI_50_). Results showed that treatment with 3.6 μM of TXA1 did not cause any major alterations in the cell cycle profile (Figure 1) but pronounced alterations in the cell cycle profile were observed with the highest concentration tested (7.2 μM). In particular, a strong increase in the sub-G1 population was observed when cells were treated with this concentration, which suggested that TXA1 induces apoptosis at this higher concentration, but not at the GI_50_ concentration.

**Figure 2:**
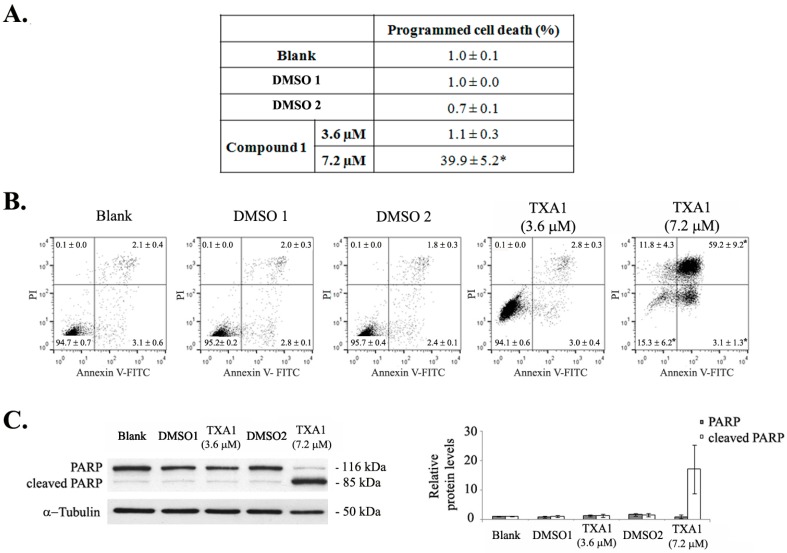
The effect of TXA1 on cell death by apoptosis of A373-C5 cells. Cells were treated for 48 h with medium (blank), TXA1 (3.6 μM and 7.2 μM), or with the corresponding DMSO concentrations (DMSO 1 and DMSO 2, respectively). (**A**) Levels of programmed cell death were analyzed with the TUNEL assay. Etoposide (1 μM) was used as a positive control (4% ± 0.1% of programmed cell death). * *p* < 0.05 Blank vs. treatment (**B**) Flow cytometry analysis of apoptotic cell death following Annexin V-FITC/PI staining. Images are representative of three independent experiments (values correspond to the mean ± SEM). Etoposide (1 μM) was used as positive control (18% ± 1.4% apoptosis); (**C**) PARP levels were analyzed by Western blot. Image is representative of 4 independent experiments (left panel). Densitometry analysis of the Western blots is expressed after normalization of the values obtained for each protein with the values obtained for tubulin (in relation to blank cells) and represent the mean ± SEM from four independent experiments (right panel).

Induction of apoptotic cell death following treatment with 7.2 μM of TXA1 (but not with 3.6 μM) was further confirmed with the Terminal deoxynucleotidyl transferase dUTP nick end labeling (TUNEL) assay (Figure 2A) and by flow cytometry analysis following Annexin V/FITC and PI staining (Figure 2B). Results from both of these assays clearly showed that treatment with 7.2 μM of TXA1 strongly induced cell death by apoptosis, although no alterations were observed following treatment with 3.6 μM TXA1 (GI_50_ concentration). These results were further corroborated by the analysis of PARP cleavage in these cells, by Western blot (Figure 2C). Indeed, a very strong increase in PARP cleavage was detected only following treatment with the highest TXA1 concentration tested (7.2 μM).

### 2.2. TXA1 Maps onto a Pharmacophore for Autophagy Induction

**Figure 3:**
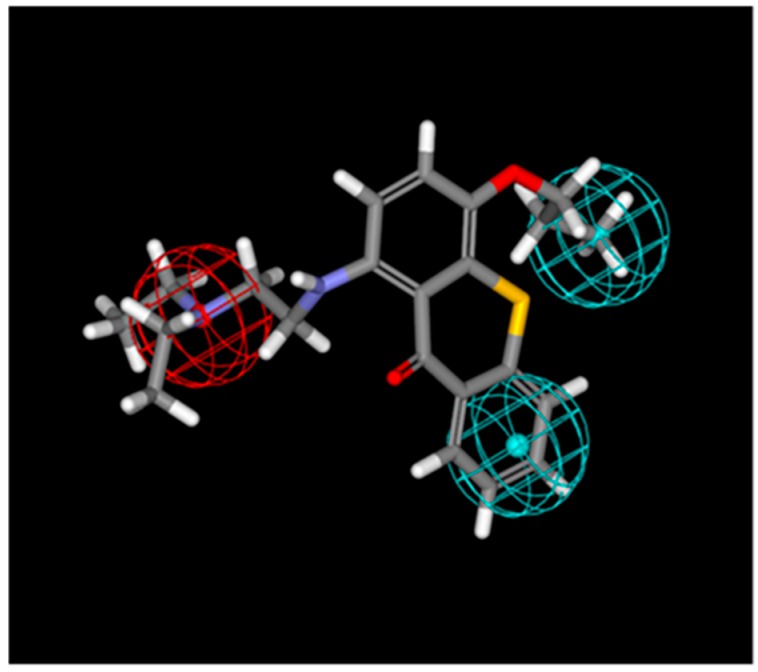
TXA1 mapped to the pharmacophore for autophagy induction. The red sphere represents the positive ionizable group and the blue sphere represents the hydrophobic region.

Given the similarity of TXA1 with *N*^10^-substituted phenoxazines (isosteres of thioxanthones which were described as autophagy inducers [26]) and since a pharmacophore had been previously developed for autophagy induction using these compounds [26], the possibility of the involvement of autophagy in the mechanism of action of TXA1 was considered using a pharmacophore approach. This was carried out using the referred pharmacophore and verifying its ability to identify autophagy inducers with different scaffolds from those it had been created from. TXA1 was mapped to the tree-feature pharmacophore (Figure 3) with results showing that TXA1 fits the pharmacophore for autophagy induction in 125 possible conformations. In all of the conformations, the nitrogen from the tertiary amine fits the positive ionizable group (red sphere on Figure 3). In 90% of the mapped conformations, one aromatic ring from the thioxanthonic scaffold fits one of the hydrophobic features, whereas the propoxyl carbonated chain fits the remaining hydrophobic feature (blue spheres on Figure 3).

### 2.3. The GI_50_ Concentration of TXA1 Induces A375-C5 Cellular Autophagy

The possible involvement of autophagy in the TXA1 mechanism of action was evaluated in vitro, in the A375-C5 cells. The GI_50_ concentration of TXA1 (3.6 μM) was selected to carry out these studies, in order to avoid alterations in apoptosis or in the cell cycle profile (observed above when using the 7.2 μM treatment but not the 3.6 μM treatment).

**Figure 4:**
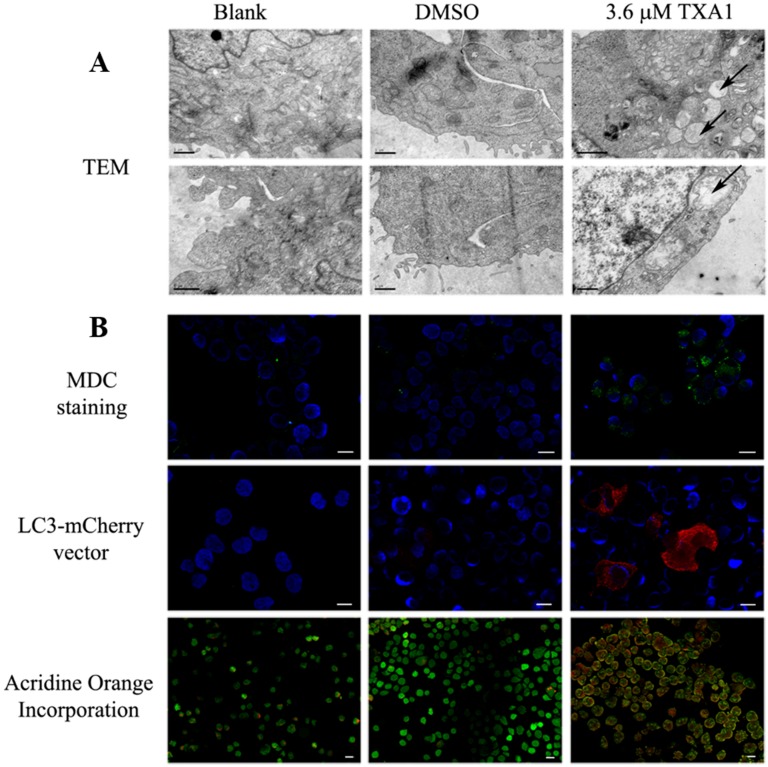
The effect of TXA1 on A375-C5 cellular autophagy. Cells were treated for 48 h with medium (blank), TXA1 (3.6 μM) or with the corresponding concentration of DMSO. (**A**) Transmission electron microscopy (TEM). Images are representative of two independent experiments. Arrows indicate autophagic structures. Bar corresponds to 1 μm; (**B**) Fluorescence microscopy analysis after (top panel) MDC staining (green); (middle panel) transfection with LC3-mCherry vector (red); and (lower panel) acridine orange incorporation (orange-red). Cell nuclei are stained with DAPI (blue). Bar = 20 μm. Images are representative of two experiments (except for MDC assay which is representative of three experiments).

The ultrastructural analysis (by transmission electron microscopy) of A375-C5 cells treated with 3.6 μM TXA1 showed the presence of autophagic structures (whereas the presence of such structures was very seldom seen in control cells) (Figure 4A). In agreement with this, MDC staining of A375-C5 cells following treatment with 3.6 μM TXA1 showed a clear punctate accumulation of MDC, indicating the presence of autophagosomes (Figure 4B). Other assays further confirmed the involvement of autophagy in the mechanism of action of TXA1. Indeed, transfection of A375-C5 cells with a mCherry-LC3 (microtubule-associated protein 1 light chain 3) expression vector allowed further visualization of the presence of LC3-II in autophagosomes, with a punctuated LC3 expression pattern being evident in transfected cells following TXA1 treatment (Figure 4B). Moreover, the formation of autophagolysosomes following TXA1 treatment was confirmed by fluorescence microscopy following staining with acridine orange. Indeed, results showed that untreated control cells presented predominantly green fluorescence with very minimal red fluorescence, whereas cells treated with TXA1 displayed considerable red fluorescence, typical of acidic vacuolar organelles (Figure 4B).

**Figure 5:**
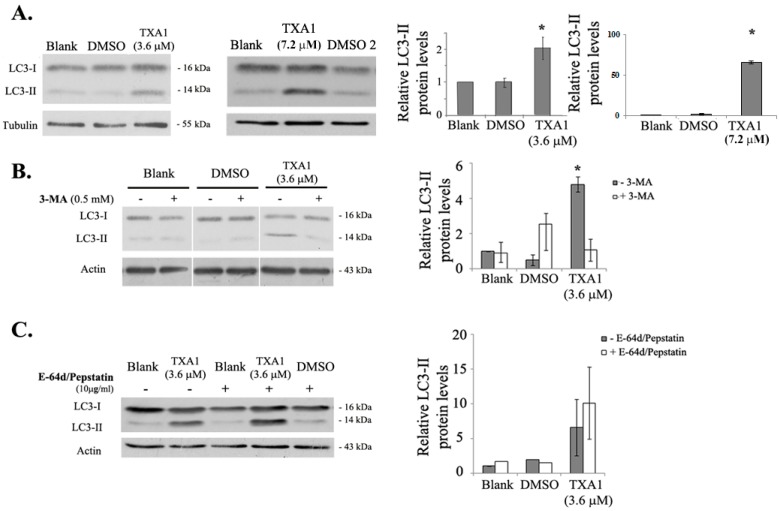
Effect of TXA1 on the expression levels of LC3-II in A375-C5 cells. Cells were treated for 48 h with medium (blank), TXA1 (3.6 μM or 7.2 μM), or with the corresponding DMSO concentrations (DMSO or DMSO2, respectively) LC3-II protein levels were analyzed by Western blot. (**A**) Following treatment with TXA1 alone; (**B**) following co-treatment with 3-MA; and (**C**) following co-treatment with E-64d/pepstatin. Images are representative of, at least, three independent experiments (except for the case of blank and DMSO treatments in the presence of E-64d/pepstatin, which result from two experiments only). Results of the densitometry analysis are expressed after normalization of the values obtained for each protein with the values obtained for tubulin or actin (and further expressed in relation to blank cells) and represent the mean ± SEM from, at least, three independent experiments (except for the case of blank and DMSO treatments in the presence of E-64d/pepstatin, which result from two experiments only). * *p* ≤ 0.05 Blank vs. treatment.

Moreover, LC3-II levels were also analyzed by Western blot (Figure 5A). Results showed an increase in LC3-II levels following treatment with 3.6 μM TXA1, further indicating that TXA1 modulates autophagy in A375-C5 cells. 

To further understand if TXA1 was an inducer or an inhibitor of autophagy, treatment with this compound was carried out in the presence of 3-methyl adenine (3-MA, a selective inhibitor of the early stages of autophagy [27]) or with E-64d/pepstatin (lysossomal protease inhibitors which inhibits autophagy at the later stage), to assess the autophagic flux [28,29,30]. Results showed that 3-MA treatment clearly reduced the levels of LC3-II induced by TXA1 (Figure 5B), supporting the idea that TXA1 was an inducer of autophagy. Furthermore, an additive increase in the levels of LC3-II was observed after co-treatment with E-64d/pepstatin, when compared to TXA1 treatment alone, showing that the autophagic flux was occurring in TXA1-treated cells. Thus, it may be concluded that TXA1 is an inducer of autophagy.

A similar autophagic effect was observed for TXA1 hydrochloride (TXA1.HCl) on a breast adenocarcinoma cell line, with increases in the autophagic structures and LC3-II levels (Supplementary Figure S2).

**Figure 6:**
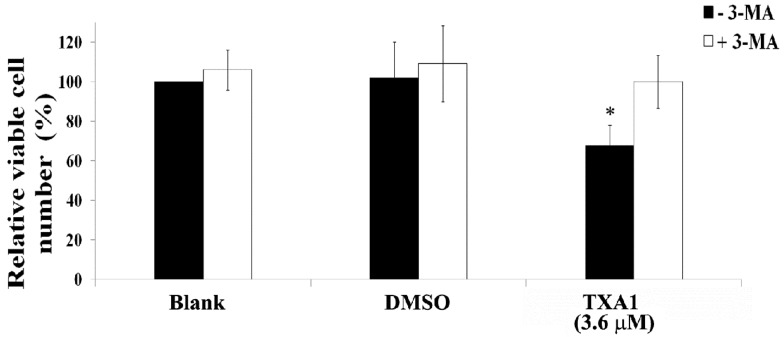
Effect of co-treating A375-C5 cells with TXA1 and 3-MA, on viable cell number. Cells were treated for 48 h with medium (blank), TXA1 (3.6 μM), or with the corresponding concentration of DMSO, in the absence or presence of 3-MA. Viable cell numbers were analyzed with a trypan blue exclusion assay. Results are presented as the percentage of viable cells in relation to blank cells and are the mean ± SE of three independent experiments. * *p* ≤ 0.05 Blank vs. treatment.

Finally, the effect of cellular co-treatment with TXA1 and 3-MA was verified on viable cell numbers, in order to confirm whether the induction of autophagy by TXA1 was responsible for the cytotoxic effect of this molecule. As expected, treatment with 3-MA alone had no effect on A375-C5 viable cell numbers (Figure 6). However, the presence of 3-MA reverted the cytotoxic effect of TXA1, proving that the cytotoxic effect of TXA1 is dependent on autophagy induction.

## 3. Discussion

Although autophagy is mainly considered a survival mechanism, there is increasing evidence that it plays dual roles in cancer, acting also as a tumor suppressor mechanism, or even as a cell death mechanism. This may depend not only on the cellular context, but also on the levels and duration of cellular autophagy [7,8,31]. Excessive or sustained autophagy has the potential to induce tumor cell death and this may explain the antitumor effect of autophagy inducers [7,8]. Indeed, several antineoplastic agents have been described to induce autophagy, leading to cell death [32]. These agents include conventional cytotoxic drugs, as well as molecularly-targeted anticancer drugs, such as imatinib [15,31], cetuximab [33], and histone deacetylase (HDAC) inhibitors [34]. Thus, there is increasing interest in the development of compounds which modulate autophagy for anticancer therapy.

We have been studying thioxanthonic molecules and derivatives, since their heterocyclic scaffold has been associated with several biological properties, including anti-parasitic, anti-oxidative, and antitumor activities [35]. In our previous studies, TXA1 has emerged as a hit thioxanthone presenting tumor cell growth inhibitory activity towards several human tumor cell lines. In addition, it has also been verified that TXA1 did not affect the growth of non-tumor MRC5 cells [23,24]. This compound presented a 2-(diethylamino)ethylamine side chain located at position 1 which was identical to the amine in the same position of lucanthone, the first thioxanthone described as a potential antitumor agent (originally used as an anti-schistosomal drug) which reached clinical trials [35,36], being currently in Phase II clinical trials for glioblastoma multiforme [37]. Moreover, TXA1 was also structurally similar to *N*^10^-substituted phenoxazines (isosteres of thioxanthones). Interestingly, autophagy modulation had been previously described for both lucanthone (described as a chemo- and radio-sensitizer associated with autophagy inhibition [25]) and for *N*^10^-substituted phenoxazines (described as inducers of autophagy [26]). Considering the structural similarity of TXA1 with the referred compounds, the present study explored the mechanism of action of TXA1 in melanoma cells, particularly regarding the modulation of autophagy. 

A promising association of TXA1 to the autophagic process resulted from the fact that TXA1 fitted a pharmacophore for autophagy induction in 125 possible conformations (Figure 3). When analyzing the effect of this compound in vitro, in A375-C5 cells, while not inducing apoptosis (or affecting cell cycle profile) at the GI_50_ concentrations, this treatment caused a clear accumulation of autophagic structures. This was observed not only by TEM (Figure 4A), which is described as a gold standard method for autophagy detection [30], but also by the increase in MDC staining and LC3 punctate pattern (Figure 4B). Autophagy modulation by TXA1 was further confirmed by the conversion of the LC3 from the cytosolic form (LC3-I) into the autophagosome-associated form (LC3-II), which is considered a marker for autophagy (observed by Western blot, Figure 5) [38,39]. Moreover, this increase in LC3-II levels following TXA1 treatment was reverted in the presence of the early-stage autophagy inhibitor 3-MA (Figure 5B).

Nonetheless, the observation of an increase in the LC3-II levels could be due to an induction of autophagy or to an inhibition of the autophagic flux. One common strategy to evaluate this is by analyzing LC3-II levels, by Western blot, in the presence and absence of lysosomal degradation inhibitors [29,30]. The fact that the presence of these inhibitors (E-64d/pepstatin) further increased LC3-II levels when compared to treatment with TXA1 alone (Figure 5C), showed that TXA1 promoted an increase in the autophagic flux. Moreover, acridine orange staining further corroborated these results by showing the presence of acidic structures, such as autophagolysosomes, in TXA1-treated cells (Figure 4B).

Finally, the cytotoxicity of TXA1 (3.6 μM) in A375-C5 cells was reverted by the inhibition of autophagy with 3-MA (Figure 6). Indeed, this compound is a widely accepted inhibitor of autophagosome formation, acting through the inhibition of class III phosphatidylinositol 3 (PI3)-kinase [8]. Several other studies have also described the use of 3-MA to investigate the involvement of autophagy in the cytotoxic effect of different molecules/natural extracts [40,41,42,43]. In the present study, in addition to the reversion of the increase in LC3 II levels induced by TXA1, pretreatment with 3-MA also rescued A375-C5 cellular viability, further supporting the role of autophagy in the cytotoxic effect of TXA1 in these cells.

## 4. Materials and Methods

### 4.1. Compound

TXA1, 1-{[2-(diethylamino)ethyl]amino}-4-propoxy-9*H*-thioxanthen-9-one, has been previously synthesized by our group as described [23]. Its purity was determined by High-Performance Liquid Chromatography with Diode-Array Detection (HPLC-DAD) analysis using an isocratic elution of MeOH/H_2_O basified with triethylamine (TEA) (1%) at a constant flow rate of 1.0 mL/min [23]. A 60 mM stock solution was prepared in DMSO and stored at −20 °C.

### 4.2. Mapping of TXA1 onto Pharmacophores for Autophagy Induction

A common feature pharmacophore model was created using HipHop module of Catalyst (Accelrys 2.1, San Diego, CA, USA) in order to reproduce a pharmacophore for autophagy induction, as previously described by Tsvetkov et al. [26]. This pharmacophore is composed of one positive ionizable group and two hydrophobic groups. TXA1 was subjected to energy minimization using HyperChem version 8.0 (Gainesville, FL, USA). The semi-empirical AM1 (Austin Model 1) [44] method with the Polak-Ribière algorithm was employed for molecular minimization [45]. The mapping of TXA1 onto the three-feature previously-described pharmacophore for autophagy induction was performed using the “Best Fit” method in Catalyst. During the flexible fitting process, conformations on TXA1 were calculated within the 20 kcal/mol energy threshold. Maximum omitted features were set to zero.

### 4.3. Cell Culture

The A375-C5 melanoma cell line (ECACC, Salisbury, UK) was genotyped at IPATIMUP´s Parentage and Genetic Identification Services Unit, using a PowerPlex^®^ 16 HS System (Promega, Fitchburg, MA, USA) according to International Society for Forensic Genetics (ISFG) guidelines. Cells were routinely cultured in RPMI-1640 with UltraGlutamine I and 25 mM 4-(2-hydroxyethyl)-1-piperazineethanesulfonic acid (HEPES) buffer (Lonza, Basel, Switzerland) supplemented with 10% FBS (Fetal Bovine Serum, PAA, Cölbe, Germany), and maintained in a humidified incubator at 37 °C with 5% CO_2_. Cell number and viability were determined with trypan blue exclusion assay.

### 4.4. Cell Treatment with TXA1

Cells were plated in six-well plates (1 × 10^5^ cells/well) and allowed to adhere for 24 h. Cells were then treated with the previously-determined GI_50_ concentration (3.6 μM) [24] and with twice that concentration (2 × GI_50_, 7.2 μM) of TXA1. Control treatments were included, such as: untreated cells (blank); cells treated with DMSO as a negative control (corresponding to the volume used for the GI_50_ (DMSO 1) or for the 2 × GI_50_ (DMSO 2) concentrations); cells treated with etoposide (Sigma-Aldrich, St. Louis, MO, USA) as a positive control for cell death and cell cycle analysis; and, finally, cells under serum starvation (i.e., without FBS), as a positive control for autophagy induction. Following the indicated time points (see Figure legends), cell number and viability were determined with the trypan blue exclusion assay and cells were further processed according to the following procedures.

### 4.5. Cell Cycle Profile

Cells were fixed in 70% ice-cold ethanol and subsequently resuspended in 0.1 mg/mL RNase A and 5 μg/mL propidium iodide in PBS for 1 h. Cellular DNA content was measured by flow cytometry using a FACSCalibur flow cytometer (BD Biosciences, Erembodegem, Belgium). The percentage of cells in the different phases of the cell cycle and in the sub-G1 peak were determined using FlowJo 7.6.5 software (Tree Star, Inc., Ashland, OR, USA) after cell debris and aggregate exclusion [23,46] and plotting at least 10,000 events per sample.

### 4.6. Programmed Cell Death

TUNEL assay was carried out using the ‘‘In situ cell death detection kit—fluorescein’’ (Roche, Boulogne-Billancourt Cedex, France) as previously described [47,48]. Briefly, cells were fixed in 4% paraformaldehyde (PFA) and cytospins were prepared. Cells were then permeabilized in ice-cold 0.1% Triton X-100 in 0.1% sodium citrate and incubated with TUNEL reaction mixture (enzyme dilution 1:20). Slides were mounted in Vectashield Mounting Media with DAPI (Vector Laboratories Inc., Burlingame, CA, USA), observed in a DM2000 fluorescence microscope (Leica, Wetzlar, Germany) and a semi-quantitative evaluation was performed by counting a minimum of 500 cells per slide. In addition, a specific assay for apoptosis was carried out using the “Human Annexin-V-FITC/PI apoptosis” kit (Bender MedSystems, Vienna, Austria) as previously described [49]. All flow cytometry analyses were performed using the FACSCalibur flow cytometer (BD Biosciences), plotting at least 10,000 events per sample and using the FlowJo 7.6.5 software (Tree Star, Inc.).

### 4.7. Expression of Apoptotic and Autophagic Proteins

Cells were lysed in Winman’s buffer (1% NP-40, 0.1 M Tris-HCl pH 8.0, 0.15 M NaCl, and 5 mM EDTA) supplemented with a protease inhibitor cocktail (Roche). Protein lysates were quantified using a DC™ Protein Assay kit (Bio-Rad, Hercules, CA, USA), according to the manufacturer’s instructions and 20 μg of protein loaded on 12% sodium dodecyl sulfate polyacrylamide gel electrophoresis (SDS-PAGE) gel [50,51]. After electrophoretic transfer into nitrocellulose membranes (Amersham, Cleveland, OH, USA), membranes were incubated with the following primary antibodies: rabbit anti-poly (ADP-ribose) polymerase PARP (H-250), (1:2000, Santa Cruz Biotechnology, Heidelberg, Germany), rabbit anti-light chain 3 B, LC3 (1:1000, Cell Signaling, Leiden, Netherlands), goat anti-actin antibody (1:2000, Santa Cruz Biotechnology), or mouse anti-tubulin antibody (1:10,000, Sigma-Aldrich), and with the corresponding secondary antibodies: goat anti-rabbit IgG-HRP (1:2000, Santa Cruz Biotechnology), donkey anti-goat IgG-HRP (1:2000, Santa Cruz Biotechnology), or goat anti-mouse IgG-HRP (1:2000, Santa Cruz Biotechnology). Signals were detected using Amersham™ ECL Western blotting detection reagents (GE Healthcare, Cleveland, OH, USA), Amersham Hyperfilm ECL (GE Healthcare) and Kodak GBX developer and fixer (Sigma-Aldrich).

### 4.8. Monodansylcadaverine (MDC) and Acridine Orange Staining

For MDC staining, studies were carried out following 48 h incubation with 3.6 μM of TXA1 (or controls). Cells were incubated for 1 h with freshly prepared MDC (50 μM) and fixed as described above. For acridine orange staining, cells were incubated with acridine orange (1 μM) for 15 min. In both cases, cytospins were prepared and mounted in Vectashield Mounting Media with DAPI (Vector Laboratories Inc., Burlingame, CA, USA). Cells were observed using a fluorescence microscope (Axio Imager.Z1 coupled with ApoTome Imaging System microscope, Zeiss, (Oberkochen, Germany).

### 4.9. Transfection with LC3-mCherry Expression Vector

Cells were plated in culture slides (Falcon; 2 × 10^4^ cells/well) and allowed to adhere for 24 h. Transfection with LC3-mCherry vector (a kind gift from T. Johansen [52]) was then carried out using lipofectamine (Invitrogen, Carlsbad, CA, USA) according to the manufacturer’s instructions. During the initial 4 h transfection, cells were incubated with medium with 5% FBS, then replaced by medium with 10% FBS [53]. Following 24 h transfection, cells were treated for 48 h with 3.6 μM of TXA1 or with controls (blank and DMSO). Cells were then fixed in 4% PFA in PBS and analyzed in a fluorescence microscope (Axio Imager.Z1 coupled with ApoTome Imaging System microscope, Zeiss).

### 4.10. Transmission Electron Microscopy

Following 48 h incubation with 3.6 μM TXA1 (or controls), cells were fixed with 2% glutaraldehyde and 2% PFA in 0.1 M phosphate buffer (PB), washed with 0.1% Millipore-filtered PB tannic acid, post-fixed with 1% PB osmium tetroxide for 1 h, and stained with 1% Millipore-filtered uranyl acetate. Samples were dehydrated in increasing concentrations of ethanol and, finally, in propylene oxide for 30 min. Samples were then infiltrated and embedded directly in Epon resin and polymerized in a 70 °C oven for two days. Cuts were performed on ultrathin (50–100 nm) sections with a Leica (Wetzlar, Germany) Ultracut microtome and each section was stained with 5% uranyl acetate solution and with Reynold’s lead citrate solution. Images were examined in a Jeol JEM 1400 transmission electron microscope (Tokyo, Japan) at an accelerating voltage of 80 kV. Digital images were obtained using a Gatan SC 1000 ORIUS CCD camera (Warrendale, PA, USA).

### 4.11. Treatment with Autophagy Inhibitors

Cells were plated in six-well plates (1 × 10^5^ cells/well) and allowed to adhere for 24 h. Cells were then treated for 1 h with 0.5 mM 3-methyladenine (3-MA, Sigma) or with 10 μg/mL E-64d (AppliChem, Darmstadt, Germany) and Pepstatin A (Cayman Chemical, Ann Arbor, MI, USA) and then co-incubated with 3.6 μM of TXA1, or with control treatments (blank, DMSO) for 48 h. Viable cell numbers were then assessed by trypan blue assay and protein expression analyzed by Western blot, as described above.

### 4.12. Statistical Analysis

Results are expressed as mean ± standard error. Values for each treatment (or solvent) were compared by Student’s *t*-test, and the differences were considered statistically significant if *p* ≤ 0.05.

## 5. Conclusions

This work reinforces the anticancer potential of the hit thioxanthonic small molecule TXA1. In particular, it shows that this compound decreases the viability of human tumor cell lines by inducing autophagy. Therefore, TXA1 may serve as lead compound for the development of new autophagy modulators with antitumor activity. Future work will allow to further elucidate the intracellular signaling cascades associated with the effect of TXA1 in autophagy, such as the phosphatidylinositol 3-kinase/mammalian target of rapamycin (PI3K/mTOR) or the AMP-activated protein kinase (AMPK) signaling pathways [11]. Furthermore, future studies with human tumor xenograft models in nude mice will allow confirmation of the in vivo lack of toxicity and antitumor potential of this hit compound.

## Supplementary Materials

Supplementary materials can be accessed at: http://www.mdpi.com/1420-3049/21/10/1343/s1.
