# Supplementary material for: Modulation of Autophagy by a Thioxanthone Decreases the Viability of Melanoma Cells
Source: Molecules. 2016 Oct 10;21(10):1343. doi: 10.3390/molecules21101343 (PMC6274546; doi:10.3390/molecules21101343)
Supplement: Supplementary file 1 [file molecules-21-01343-s001.pdf]

Supplementary Materials: Modulation of Autophagy by a Thioxanthone Decreases the Viability of Melanoma Cells

Raquel T. Lima, Diana Sousa, Ana M. Paiva, Andreia Palmeira, João Barbosa, Madalena Pedro, Madalena M. Pinto, Emília Sousa and M. Helena Vasconcelos

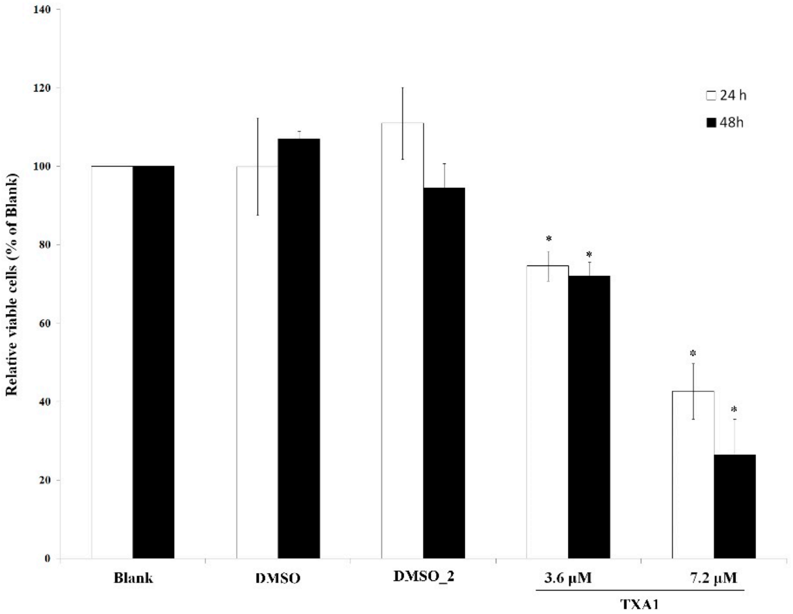

Figure S1. Treatment of A375-C5 cells with TXA1 reduced the viable cell number. Cells were treated for 48 h with medium (Blank), TXA1 (3.6 µM and 7.2 µM), or with the corresponding DMSO concentrations (DMSO and DMSO 2, respectively). Results are presented as the percentage of viable cells in relation to Blank cells (treated with medium only) and are the mean ± SE of four independent experiments (except for results from the 48 h treatment with the lowest DMSO control treatment, which are the mean of three experiments only). * $p < 0.05$ Blank vs. treatment.

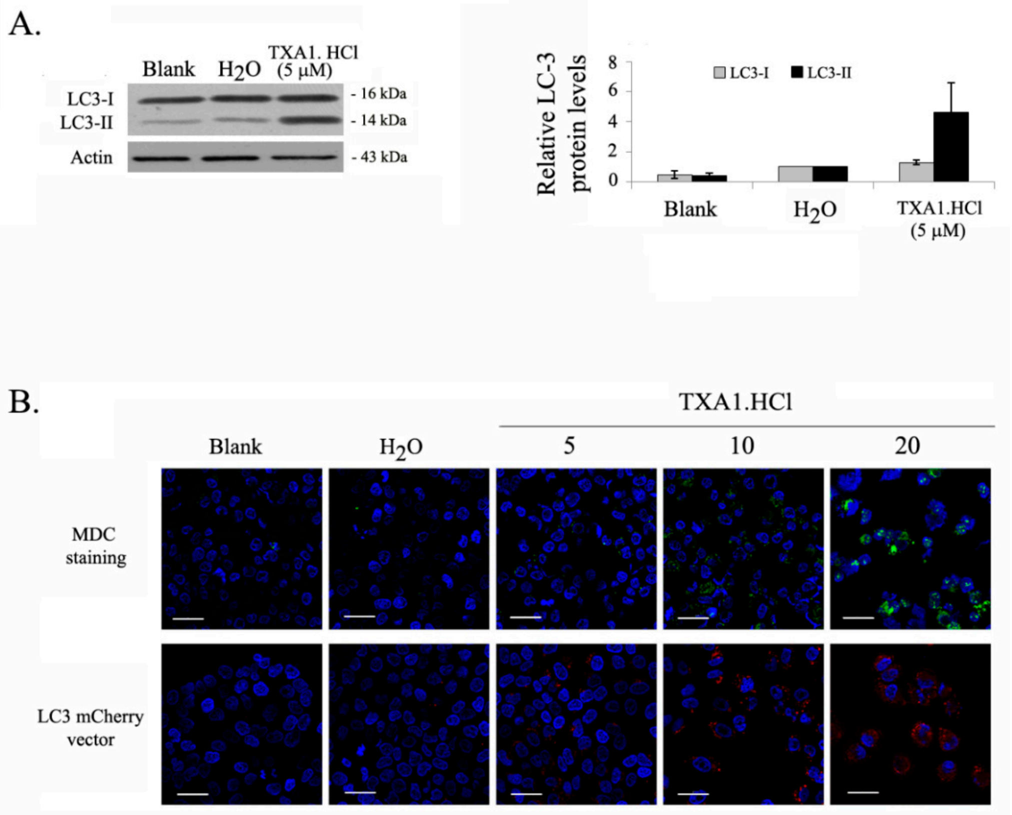

Figure S2. TXA1.HCl induces autophagy in breast adenocarcinoma MCF7 cells. (**A**) Representative Western blot showing increased LC3-II levels in cells treated for 48 h with medium (Blank), TXA1.HCl (the hydrosoluble salt of TXA1 [24]), or the corresponding solvent (H_2O). Results of the densitometry analysis are expressed after normalization of the values obtained for each protein with the values obtained for actin (and further expressed in relation to solvent treatment) and represent the mean ± SEM from three independent experiments; (**B**) MDC staining of cells treated with medium (Blank), H_2O (control), or with 5, 10, or 20 µM of TXA1.HCl, showing the presence of autophagic structures analyzed by fluorescence microscopy. Upper panel: MDC staining (green); lower panel: transfection with LC3-mCherry vector (red). Cell nuclei are stained with DAPI (blue). Bar = 20 µm. Images are representative of three experiments (except for MDC assay which is representative of two experiments).
